# Supplementary material for: Molecular Pharmacology of Gelsemium Alkaloids on Inhibitory Receptors
Source: Int J Mol Sci. 2024 Mar 16;25(6):3390. doi: 10.3390/ijms25063390 (PMC10970644; doi:10.3390/ijms25063390)
Supplement: Supplementary file 1 [file ijms-25-03390-s001.zip › ijms-2868549-supplementary.pdf]

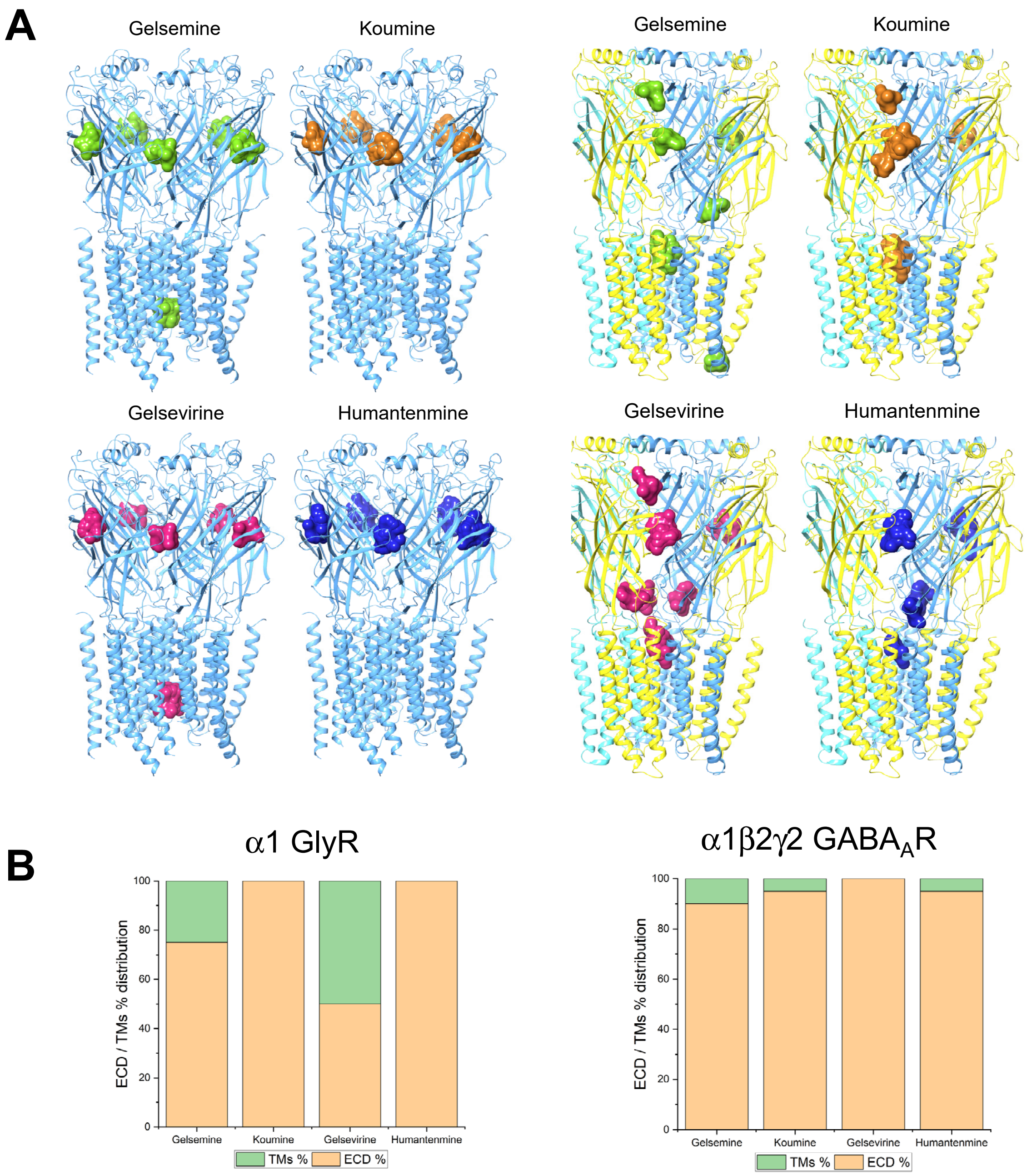

Supplementary Figure S1. Molecular sites for Gelsemium alkaloids on homomeric α1GlyRs and on α1β2γ2 GABA$_A$Rs. A. Structural models of the putative interactions of gelsemine (green),

koumine (orange), gelsevirine (pink), and humantenmine (blue) with GlyRs and $GABA_A$Rs. The α1GlyR subunits are showed in blue. For $GABA_A$Rs, α1 subunits are shown in yellow, β2 subunits in cyan and γ2 subunits in blue. B. The graph summarizes the percentage of alkaloid-receptor complexes formed in the extracellular domain (ECD) and in the transmembrane domains (TM). Structures of α1GlyRs (PDB:1TU9) and of α1β2γ2 $GABA_A$Rs (PDB:6X3S) were used. The docking study was performed using Autodock Vina and GLIDE programs.

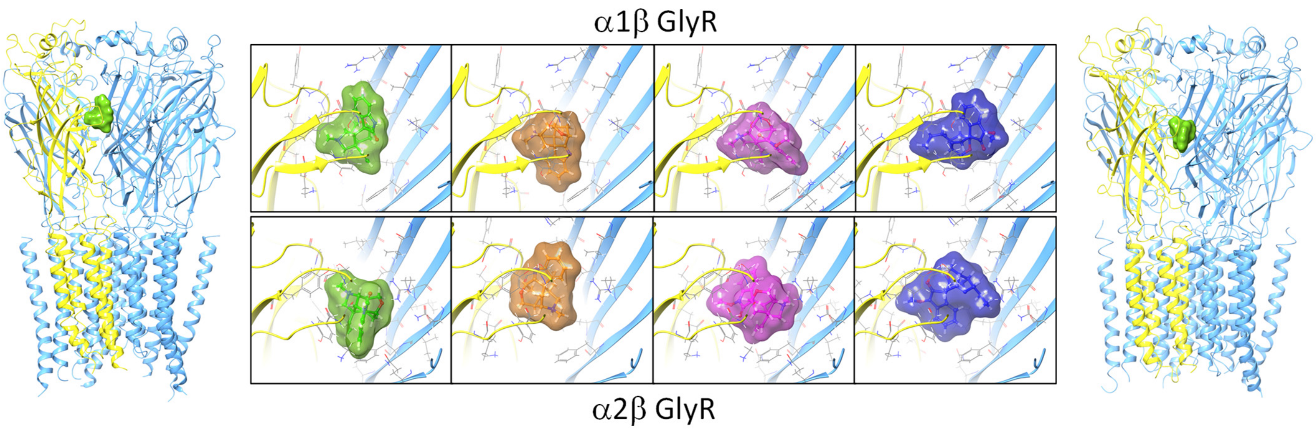

Supplementary Figure S2. Putative binding of Gelsemium alkaloids to the α–β interphase of heteromeric GlyR subunits. The central panels describe the binding of gelsemine (green), koumine (orange), gelsevirine (magenta), and humantenmine (blue) to the α–β interphase of heteropentameric α1βGlyRs (upper panels) and α2βGlyRs (lower panels). The left (α1β) and right (α2β) models show and lateral view of the pentameric structures bound to gelsemine. The docking scores for the interaction of gelsemine, koumine, gelsevirine and humantenmine to the α–β interphase were similar to the orthosteric sites values (α/α interphase).

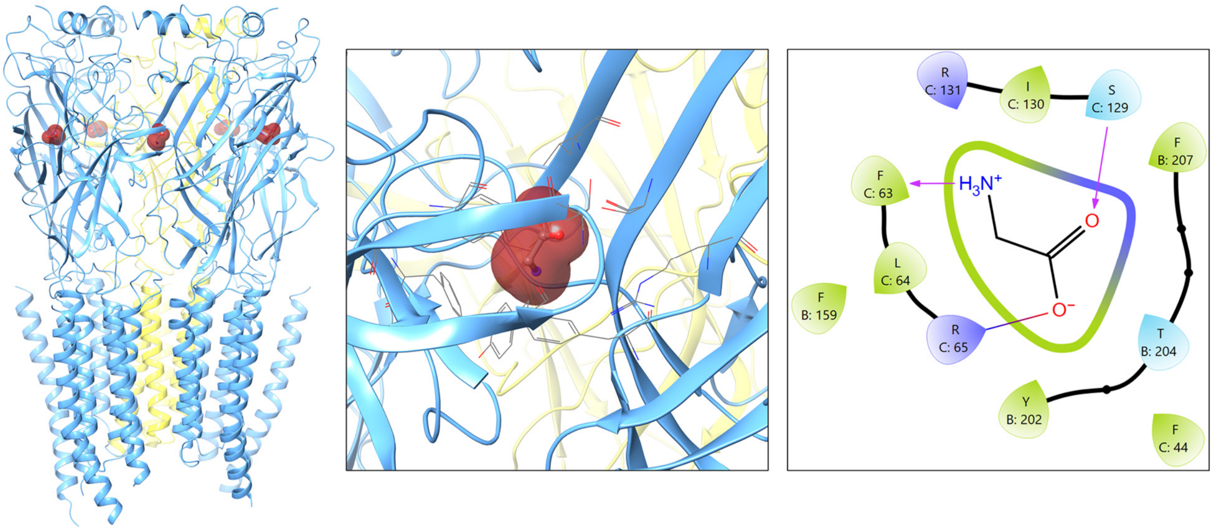

Supplementary Figure S3. Interaction of glycine at the orthosteric site of the α1 GlyR. Utilizing a redocking strategy employing the open conformation structure of human GlyR α1β (PDB ID 8ND5), we have reconstructed the receptor-agonist complex. The left and center panels depict the localization of five glycine molecules bound to the orthosteric site, accompanied by respective zoomed images of an α/α interface, highlighting the side chains of amino acids within proximity of 4Å from the glycine. The right panel presents an interaction scheme detailing the amino acids and interactions responsible for stabilizing the glycine binding. Purple arrows denote hydrogen bonds, while the red-blue lines represent salt bridge interactions. Numbered residues are illustrated by colored markers, with the color code indicating amino acid properties (green for hydrophobic residues, blue for positively charged residues, and cyan for polar residues).

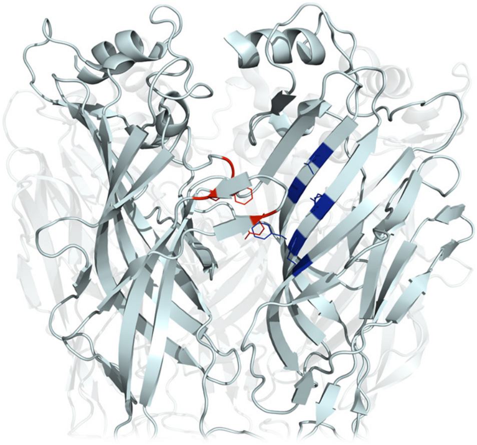

```
                                                     44
                                                     ♦
α1 GlyR   -------ARSAPKPMSPSDFLDKLMGRTSGYDARIRPNFKGPPVNVSCNIFINSFGSIAE   53
α2 GlyR   KDHDSRSGKQPSQTLSPSDFLDKLMGRTSGYDARIRPNFKGPPVNVTCNIFINSFGSVTE   60
α3 GlyR   -------ARSRSAPMSPSDFLDKLMGRTSGYDARIRPNFKGPPVNVTCNIFINSFGSIAE   53
                 .:.     :******************************:**********::*

                   63 65
                   ♦♦♦
α1 GlyR   TTMDYRVNIFLRQQWNDPRLAYNEYPDDSLDLDPSMLDSIWKPDLFFANEKGAHFHEITT   113
α2 GlyR   TTMDYRVNIFLRQQWNDSRLAYSEYPDDSLDLDPSMLDSIWKPDLFFANEKGANFHDVTT   120
α3 GlyR   TTMDYRVNIFLRQKWNDPRLAYSEYPDDSLDLDPSMLDSIWKPDLFFANEKGANFHEVTT   113
          *************:*** ****.******************************:**::**

                      127 129                             159 160
                       ♦   ♦                               ▼▼
α1 GlyR   DNKLLRISRNGNVLYSIRITLTLACPMDLKNFPMDVQTCIMQLESFGYTMNDLIFEWQEQ   173
α2 GlyR   DNKLLRISKNGKVLYSIRLTLTLSCPMDLKNFPMDVQTCTMQLESFGYTMNDLIFEWLSD   180
α3 GlyR   DNKLLRIFKNGNVLYSIRLTLTLSCPMDLKNFPMDVQTCIMQLESFGYTMNDLIFEWQDE   173
          ******* :**:******:****:*************** ***************** .:

                                         202  207
                                          ▼    ▼
α1 GlyR   GAVQVADGLTLPQFILKEEKDLRYCTKHYNTGKFTCIEARFHLERQMGY   222
α2 GlyR   GPVQVAEGLTLPQFILKEEKELGYCTKHYNTGKFTCIEVKFHLERQMGY   229
α3 GlyR   APVQVAEGLTLPQFLLKEEKDLRYCTKHYNTGKFTCIEVRFHLERQMGY   222
          . ****:*******:*****:* ***************.:*********

▼ Principal subunit   ♦ Complementary subunit
```

Supplementary Figure S4. Primary sequence alignment of the extracellular domain of α1, α2 and α3 GlyRs. The key residues involved in the interaction between gelsemine, koumine and gelsevirine with GlyRs are fully conserved and are highlighted in blue (complementary subunit) and in the red (principal subunit). The left ribbon model highlights the localization of the residues on both subunits.
